# BAF Complex in Embryonic Stem Cells and Early Embryonic Development

**DOI:** 10.1155/2021/6668866

**Published:** 2021-01-16

**Authors:** Heyao Zhang, Xuepeng Wang, Jingsheng Li, Ronghua Shi, Ying Ye

**Affiliations:** ^1^Cam-Su Genomic Resource Center, Medical College of Soochow University, Suzhou, Jiangsu 215000, China; ^2^College of Traditional Chinese Medicine, Macau University of Science and Technology, China; ^3^School of Life Sciences, University of Science and Technology of China, China

## Abstract

Embryonic stem cells (ESCs) can self-renew indefinitely and maintain their pluripotency status. The pluripotency gene regulatory network is critical in controlling these properties and particularly chromatin remodeling complexes. In this review, we summarize the research progresses of the functional and mechanistic studies of BAF complex in mouse ESCs and early embryonic development. A discussion of the mechanistic bases underlying the distinct phenotypes upon the deletion of different BAF subunits in ESCs and embryos will be highlighted.

## 1. Introduction

Embryonic stem cells (ESCs) are derived from the inner cell mass of blastocysts in early embryos [1–3]. With the remarkable abilities to indefinitely self-renew and differentiate to all types of cells in the body, ESCs become an ideal model to study cell fate determination and lineage differentiation, therefore having broad applications in the fields of regenerative medicine and translational medicine.

Since their isolation, the mechanism underlying the self-renewal and pluripotency of ESCs has been the focus of intensive research in the field of stem cell biology [4]. Numerous studies demonstrate that the identity of ESCs is controlled by a core transcriptional regulatory network composed of signaling pathways such as the LIF/STAT3 pathway [4–6], pluripotent transcription factors such as OCT4, SOX2, NANOG, and KLF4 [7–9], protein complexes [10–12], microRNAs [13], and chromatin remodeling complexes [12].

## 2. Chromatin Remodeling Complexes

Specific transcriptomes expressed in different types of mammalian cells are controlled partly by their unique chromatin states. The regulation of chromatin states selectively causes gene expression or silencing via controlling the access of transcriptional factors to gene regulatory elements. This variation of transcriptional activity according to the chromatin structural changes is called chromatin remodeling [14]. There are two main types of chromatin remodeling: one is covalent histone modification, which includes acetylation, methylation, phosphorylation, and ubiquitination; the other is ATP-dependent physical modification, which is achieved mainly through ATP-dependent protein complexes [14].

**Figure 1:**
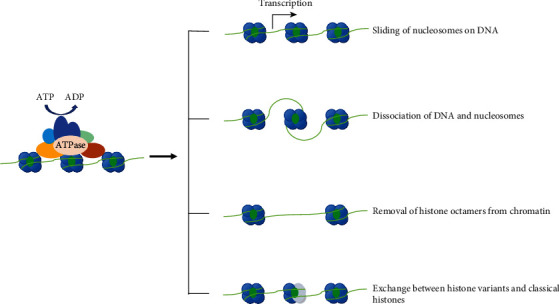
Schematic of the chromatin remodeling complex functional mode. Chromatin remodeling complex, which has ATPase activity, could change the structure of nucleosomes with the energy generated by hydrolyzing ATP, to regulate the accessibility of chromatin and further affect gene expression.

The ATP-dependent protein complexes with ATPase activity, termed chromatin remodeling complex, use the energy generated by hydrolysis of ATP to make the four changes in the nucleosomes structure and thereby regulate gene expression (Figure 1) [15].

According to the difference in structure and composition of ATPase, chromatin remodeling complexes are divided into four categories: switching (SWI)/sucrose nonfermenting (SNF) [16, 17], INO80 [18], ISWI (imitation SWI) [19], and CHD (chromodomain helicase DNA binding) [20].

## 3. Structure and Function of SWI/SNF

**Figure 2:**
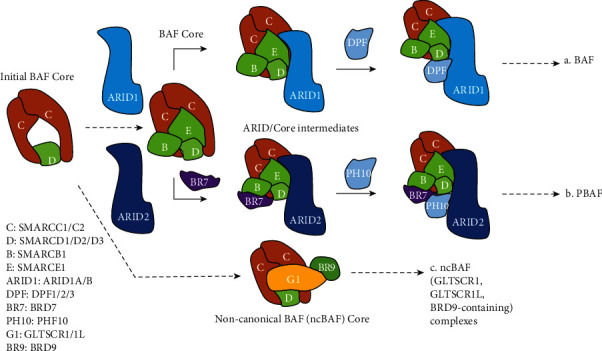
Three types of SWI/SNF assembly processes. (a) BAF: ARID1 and BAF core modules form a subcomplex (ARID1/BAF core), then combine with DPF2, and recruit ATPase modules (including SS18) to complete BAF assembly; (b) PBAF: ARID2 first combines with BAF core to form a subcomplex (ARID2/PBAF core), then combines BRD7 and PHF10, followed by the recruitment of ATPase module (excluding SS18), and finally combines with PBRM1 to complete PBAF assembly; (c) ncBAF: GLTSCAR1/1L BRD9 combines with BAF core module to form the core module of ncBAF and combines BRD9 with ATPase module (containing SS18) to form the ncBAF complex [25, 26].

The SWI/SNF complex is first discovered in yeast [16] and later in Drosophila [21] and mammals [22, 23]. The mammalian SWI/SNF complex, also named BAF (BRG1/BRM-associated factor) complex, is a multi-subunit protein complex of about 2 MDa, which is composed of 12-15 subunits encoded by 29 genes [24]. According to the different composition of subunits, BAF complexes are divided into canonical BAF (cBAF), PBAF, and noncanonical (ncBAF) [25]. The structural characteristics of the three types of SWI/SNF complexes are shown in Figure 2. Recent studies reveal the assembly process of these three types of BAF complexes (Figure 2) [25, 26] In different developmental stages and different tissues, the composition of the BAF complex also changes to regulate distinct gene expression, thereby performing different functions [27].

## 4. The Role of BAF Complex in mESCs

**Figure 3:**
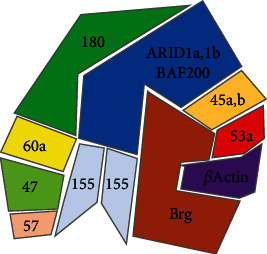
The subunits constituent of the esBAF complex.

esBAF, a specific BAF complex in ESCs, consists of 9-11 subunits, which includes the ATPase subunit BRG1 not BRM, BAF250a instead of BAF200, BAF60a/b instead of BAF60c, and BAF155 dimer instead of BAF155 and BAF170 (Figure 3) [28]. Numerous studies reveal the functional importance of the BAF complex in ESCs and embryonic development [28–30]. Here, we summarize the roles of various subunits of the esBAF complex in ESCs (Table 1) and embryonic development (Table 2).

### 4.1. BRG1

As the core catalytic subunit of the esBAF complex, BRG1 alone can reshape nucleosomes in vitro, but the efficiency is very low. The smallest complex of four subunits, BAF155, BAF170, Baf47, and BRG1, can exert catalytic activity efficiently [44].

BRG1 participates in chromatin remodeling to maintain ESC self-renewal and pluripotency [28, 31]. The absence of Brg1 results in the impairment of ESC self-renewal and pluripotency [28, 31, 32]. Deletion of Brg1 leads to the decreased expression of Oct4 and Sox2 and increased expression of lineage-specific genes, indicating its function in ESC differentiation [28, 31]. The BRG1 null embryos die at the blastocyst stage. ES cells cannot be isolated from Brg1-deficient embryos [40, 45].

BRG1 directly binds the promoter regions of Oct4, Sox2, and Nanog genes, indicating its regulatory roles on the expression of core pluripotency genes. Consistently, BRG1 interacts directly with NANOG, OCT4, and SOX2 and binds with many of their common target genes [30, 46].

In addition, BRG1 also regulates the expression of ESC-related genes by participating in LIF/STAT3 signaling pathways [47]. Leukemia Inhibitory Factor (LIF) is required to maintain the pluripotency of mESCs and naïve human ESCs [5, 6, 48]. In mESCs, the binding of BRG1 and STAT3 colocalize extensively on the genome [30, 47]. The binding of STAT3 to genes associated with pluripotency depends on the presence of the catalytic subunit BRG1 in the esBAF complex, which loosens the chromatin structure at the target gene of STAT3 and thus responds to the LIF signal [47]. BRG1 can enhance the LIF-STAT3 signaling pathway by antagonizing the PcG complex [47]. On the other hand, BRG1 and the PRC complex bind together to four Hox loci, thereby inhibiting the differentiation of ESCs [47].

Recently, YY1 was reported to interact with BRG1 to promote proliferation and pluripotency of mouse ESCs. The knockdown of Yy1 gene downregulates Nanog and upregulates differentiation marker genes Pax3 and Cdx2 [49].

The interaction between BRG1 and TOP2 is required for the initial stage of accessibility induction. Top2 can make the chromatin more accessible for chromatin remodelers as well as transcription factors [50], suggesting that TOP2 may work together with the BAF complex to remodel chromatin and optimize BAF-mediated recruitment of transcriptional factors.

### 4.2. BAF47

BAF47 (also known as SMARCB1/SNF5/INI1) is involved in the differentiation of stem cells. The knockdown of BAF47 enhances cell pluripotency and prevents differentiation [33]. Overexpressing BAF47 promotes ESC differentiation. BAF47 can fine-tune the level of OCT4 and affect the nucleosome occupation at the regulatory region of OCT4 target genes, thus breaking the balance between pluripotency and differentiation and determining the fate of cells [33]. In contrast, a recent report indicates the upregulated Cdx2 expression in Baf47 KO ESCs [34]. Therefore, further study to clarify the function of Baf47 in ESCs is needed.

The BAF47 null blastocysts do not hatch and cannot implant into the uterus for further development [41, 42], which may cause death of Baf47 null embryos during implantation [41, 42].

### 4.3. BAF155 and BAF170

BAF155 (also known as SRG3) shares 61.7% amino acid homology with BAF170, but they have different functions [28]. The esBAF complex contains a homodimer of two BAF155 without BAF170 [28]. The deletion of BAF155 resulted in the defects of ESC self-renewal and pluripotency [28]. As expected, overexpression of BAF170 cannot restore the defects of Brg155 KO ESCs [28]. Similarly, knockdown of Baf155 expression also resulted in inhibited ESC proliferation, decreased expression of the pluripotent gene Oct4, and increased apoptosis [28]. Consistently, deletion of BAF155 fails to form inner cell mass [51].

In contrast to mESCs, esBAF in hESCs contains heterodimers composed of BAF155 and BAF170. The contents of BAF155 and BAF170 in the BAF complex seem to determine the fate of the cell [52].

The deletion of BAF155 prevented mouse embryos from developing properly and died during implantation [29]. Depletion of BAF155 leads to increased expression of Nanog in the ICM and its ectopic expression in TE. However, the overexpression of BAF155 leads to the development arrested at the E3.5 to E4.5 transition and upregulation of Cdx2 and Sox17 at E4.5 embryos [29].

### 4.4. BAF53a

BAF53a (also known as ACTL6a or ARP4) is expressed in a variety of stem/progenitor cells, including neural progenitor cells, hematopoietic stem cells, epidermal progenitor cells, and ES cells [37, 38, 53]. The knockdown of BAF53a in ESCs reduces the expression of pluripotent genes such as Oct4 and Nanog and induces ESC differentiation towards the original endoderm [37]. It is interesting that another report indicates that knockout of Baf53a increases the expression of Oct4 and Nanog. Deletion of Baf53a repressed cell proliferation and induced apoptosis [54].

### 4.5. BAF45

BAF45 has two PHD domains, which can recruit the BAF complex to specific histone modification sites [55]. BAF45 includes four subunits: BAF45a, BAF45b, BAF45c, and BAF45d [30]. Only BAF45a and BAF45d are contained in esBAF [30]. BAF45a plays an important role in the maintenance of hematopoietic stem cells [56], but its role in mESCs is not clear. BAF45d, also known as Dpf2, is widely expressed in a variety of cells [30]. Deletion of Dpf2 in mESCs leads to the differentiation defects, which cannot be restored by BAF45a and BAF45c [30]. Further study demonstrates that Dpf2 regulates ESC differentiation by regulating Tbx3 expression [30].

### 4.6. BAF250a

BAF250a (ARID1A) is a unique subunit of esBAF, which belongs to the trithorax group (TrxG) family [57, 58]. BAF250a is abundantly expressed in early mouse embryos and ESCs [35, 36]. Deletion of BAF250a inhibits ESC self-renewal and upregulates the expression of the primitive endoderm marker genes in ESCs [35, 36]. The lack of BAF250a prevents ESCs from developing into mesoderm-derived cardiomyocytes, adipocytes, and skeletal muscle cells, but can differentiate into ectoderm-derived nerve cells [35, 43].

BAF250a is necessary for the development of early embryos. The loss of BAF250a caused the development of early embryos (E6.5) of mice to stagnate, and the lack of mesoderm prevented further development of gastrulation embryos [35].

### 4.7. ncBAF in ESCs

Gatchalian and colleagues found the existence of ncBAF in mESCs that puts BRD9 as the core [39]. Compared with esBAF, ncBAF lacks BAF47, BAF57, and ARID1A subunits. The knockdown of BRD9, the core subunit of the ncBAF complex, inhibited the proliferation of ESCs [39]. Although both esBAF and ncBAF are involved in ESC self-renewal and pluripotency maintenance, ChIP-seq analysis showed that esBAF and ncBAF complexes target distinct sites in the genome and cobound with different pluripotent transcription factors (TFs) [30, 39, 45]. esBAF tends to bind to active enhancers rich in h3k4me1 modification [30], while ncBAF is more likely to bind to promoter regions rich in h3k4me3 [39]. Different from esBAF, ncBAF tends to cobind with KLF4 and CTCF, indicating its distinct mechanisms from cBAF in ESCs [39].

In summary, different components of the BAF complex function differentially in ESC maintenance and differentiation. Deletion of core subunits such as Brg1, Baf155, or Baf250a reduced the expression of Oct4, Sox2, and Nanog, the key pluripotency genes of ES cells [28, 31]. On the contrary, Baf47 negatively regulated Oct4 expression in ESCs [33]. Deletion of Baf250a promoted the expression of endoderm marker genes Gata4 and Gata6 [35], while deletion of Baf45d decreased Tbx3 expression and impaired mesoendoderm differentiation [30]. During embryonic development, knockout of Brg1, Baf155, or Baf250a led to embryo death during peri-implantation [40–42, 51]. Deletion of Baf250a resulted in embryo death in later embryonic development stage [35].

Consistently, BAF complexes also play important roles in the reprogramming of somatic cells to induced pluripotent stem cells (iPSCs). Depletion of Brg1 leads to the failures in reprogramming [59, 60]. Overexpression of Brg1 and Baf155 increases the reprogramming efficiency of MEFs to iPSCs [61], whereas downregulation of Brm and Baf170 improves reprogramming efficiency [62]. Therefore, similar to the distinct roles of different BAF subunits for the maintenance and differentiation of ES cells, different BAF components also play different roles in the reprogramming.

## 5. Conclusion

BAF complexes are functionally important for the self-renewal and development of ESCs and mouse embryonic development. Deletion of different subunits in ESCs and embryos results in distinct phenotypes in ESC maintenance and differentiation and embryonic development, while the underlying mechanisms are far from clear. Schick et al.'s work reveals that the loss of a single subunit of the BAF complex did not destroy the entire complex, but will change the composition of the BAF complex [24]. Consistently, a recent study shows that deletion of Dpf2 only affects about 8% of BRG1 binding sites on the genome [30]. Therefore, it is attractive to propose that distinct BAF subunit controls the integrity of a part of the BAF complex on the genome, and therefore, its deletion only affects the binding of a part of the BAF complex, which directly changes the expression of distinct pluripotency TFs in both ESCs and differentiating cells with other TFs and chromatin modifiers. It is intriguing to extend the proposed mechanism further to other chromatin remodeling complexes. To confirm the proposal, future works are required to study the deletion of specific subunits on the binding of BRG1 and some other core factors of BAF and other chromatin remodeling complexes.

## Figures and Tables

**Table 1:** The role of BAF subunits in mESCs.

Subunit	Phenotypes	References
BRG1	Knockdown or knockout of Brg1 resulted in ESC differentiation and downregulation of self-renewal and pluripotency genes such as Oct4 and Sox2.	[28, 31, 32]
BAF47	Knockdown of Baf47 blocks differentiation; overexpression of Baf47 enhances differentiation; knockdown of Baf57 upregulates Cdx2 expression.	[33, 34]
BAF155	Depletion of BAF155 resulted in decreased proliferation, decreased Oct4 expression, and increased apoptosis of ESCs.	[28]
BAF250a	The self-renewal ability of mESCs decreases after knocking out BAF250a, and the differentiation of ES cells into the mesoderm and endoderm is inhibited.	[35, 36]
BAF45d	Knockout of BAF45d perturbs ESC self-renewal and impairs its differentiation to three lineages.	[30]
BAF53a	Knockdown of Baf53a reduces the expression of pluripotent genes in ESCs. Baf53a protects mESCs from differentiating into primitive endoderm; knockout of Baf53a represses cell proliferation and induces cell apoptosis.	[37, 38]
BRD9	Preserving the naive pluripotency of ESCs	[39]

**Table 2:** The role of BAF subunits in early mouse embryonic development.

Subunit	Phenotypes	References
BRG1	Brg1 null embryos die during implantation, and mice heterozygous for Brg1 are prone to cause tumor formation and anencephaly.	[32, 40]
BAF47	Baf47 null mice die during embryo implantation. Baf47 heterozygous mice are prone to cause anencephaly.	[41, 42]
BAF155	Baf155 knockout embryos are lethal during implantation.	[29, 34]
BAF250a	Baf250a knockout embryos die on E6.5. Baf250a regulates heart development.	[35, 43]
